# Do extreme temperatures affect cognition? A short review of the impact of acute heat stress on cognitive performance of firefighters

**DOI:** 10.3389/fpsyg.2023.1270898

**Published:** 2024-01-11

**Authors:** Catherine Thompson, Lucy Ferrie, Stephen J. Pearson, Brian Highlands, Martyn J. Matthews

**Affiliations:** ^1^Department of Psychology, Liverpool Hope University, Liverpool, United Kingdom; ^2^School of Health Sciences, University of Manchester, Manchester, United Kingdom; ^3^School of Health Sciences, University of Salford, Salford, United Kingdom

**Keywords:** cognition, firefighters, heat stress, maximal adaptability model, core body temperature, vigilance

## Abstract

Research shows that exposure to high environmental temperatures can affect task performance. Theoretical explanations outline that heat is a source of stress that competes for limited-capacity resources, therefore if a task is resource-intensive, and/or if heat stress is extreme, performance will suffer. One occupation in which individuals complete demanding tasks and make difficult decisions, often in temperatures exceeding 200°C, is firefighting. Yet very little is currently known about the impact of heat stress on the cognitive functioning of firefighters. This short review summarizes the limited research in this area, focusing on studies that measured cognition of firefighters following a realistic training exercise. The findings are mixed with evidence that heat stress improves, impairs, and has no impact on cognitive functioning. While there are differences in the firefighting activities utilized, and the temperatures that participants were exposed to, it is argued that the varied findings can be attributed to the tasks used to assess cognitive processing, and the cognitive functions being measured. In accordance with the wider field of research, it is concluded that complex functioning, such as sustained attention, vigilance, and working memory is negatively impacted by acute exposure to extreme heat. Greater understanding of factors affecting cognition would inform safety practices and more research is needed to understand how and when heat stress may influence cognition in firefighting scenarios.

## 1 Introduction

In search and rescue operations the role of a firefighter is cognitively demanding, requiring vigilance, memory for spatial locations, and rapid decision-making. This involves the use of limited-capacity resources such as sustained attention and working memory. Cognitive ability in such scenarios is impacted by experience and expertise, but may also be influenced by stressors, including complexity of the rescue task and emotional load of the situation. Hermans et al. (2014) proposed that acute stress affects cognition by reducing activity in the prefrontal cortex (an area associated with sustained attention and working memory, e.g., Eichenbaum and Cohen, 2008; Kim et al., 2017) to enable increased activity in brain areas responsible for affective processing (e.g., the amygdala).

One source of stress relatively unique to firefighting is heat stress. Studies have shown that task performance in the workplace can suffer when environmental temperatures exceed 23°C (e.g., Ramsey et al., 1983; Cheung et al., 2016), and the working environment of a firefighter often exceeds 200°C (Willi et al., 2016). Additionally, Schmit et al. (2017) concluded that cognitive function suffers when core body temperature increases beyond 39°C and core body temperature when firefighting often exceeds 38.5°C (Horn et al., 2017). This suggests that firefighters may be at-risk of heat stress, ultimately impacting their ability to protect lives. It is therefore important to understand how heat can affect firefighter cognition.

Studies measuring the effects of heat on cognition show mixed findings. Seppänen et al. (2006) found that task performance of office workers is best at 22°C but deteriorates as temperatures rise above 23–24°C. However, when testing performance of trainee surgeons, Berg et al. (2015) found no impairment when working in 26°C heat. Ashworth et al. (2021) also found no effect of temperature on cognition when participants walked on a treadmill in 33°C heat. In contrast, Liu et al. (2013) found impairments to executive control after participants spent 45-min in a chamber heated to 50°C (compared to 28°C) and Saini et al. (2017) found that sustained attention and executive functioning of soldiers working in desert conditions was worse in June (42–43°C) compared to March (24–27°C).

These findings indicate that more extreme temperatures have a greater impact on cognition. Yet, the relationship is more complex. Hancock and Vasmatzidis (2003) suggest that the effect of heat on cognition varies depending on factors such as expertise and duration of exposure, however they argue that the key factor is task complexity. In accordance with the Maximal Adaptability Model (Hancock and Warm, 1989) they proposed that stressors compete for limited-capacity cognitive resources. Individuals can adapt to this, for example by devoting more attention to a task, but as complex tasks utilize more resources, the ability to compensate reduces, meaning that stressors (i.e., heat) will impact complex tasks more than simple tasks. This is potentially illustrated by Berg et al. (2015) who found that while heat did not affect cognition, participants reported increased cognitive load and distraction suggesting they were expending more effort to maintain performance.

Hancock and Vasmatzidis (2003) concluded that demanding tasks, such as vigilance and monitoring, are most vulnerable to heat stress, and this is concerning given the importance of such tasks in firefighting, the complexity of situations a firefighter may be exposed to, and the difficult decisions they are required to make. Yet it is unclear how heat affects firefighter cognition because past research does not represent the working conditions faced by firefighters. To better understand the risks of acute heat stress on firefighter cognition, this review outlines seven studies (summarized in Table 1) that tested the effects of heat on firefighter cognition. All met the criteria of using real-life firefighting scenarios and active firefighters as participants.

**Table 1:** A summary of the methods and findings of the studies that have investigated the effects of heat on firefighter cognition using live-fire training activities and firefighters as participants.

Authors (and date)	Activity (and duration)	Sample size	Maximum environmental temperature	Maximum body temperature	Cognitive function and task	Outcome
Greenlee et al. (2014)	Live-fire training exercise indoors (18 min)	20	82°C	38.2°C (Core body temperature)	Visual continuous performance test (CPT) to measure sustained attention	Improved performance
Walker et al. (2015)	Search and rescue scenarios inside a purpose-built heat chamber (2 × 20 min)	77	110°C	41°C (Core body temperature)	Tests to measure speed of processing, vigilance, working memory	Improved vigilance
Hemmatjo et al. (2017)	Firefighting tasks in a smoke-diving room (~30 min)	17	37°C	38.32°C (Tympanic temperature)	Paced auditory serial addition task (PASAT) to measure information processing and working memory	Impaired performance
Zare et al. (2018)	Live-fire suppression outdoors (~20 min)	18	No data	38.07°C (Temporal artery temperature)	PASAT to measure information processing and working memory	Impaired performance
Hemmatjo et al. (2020)	Live-fire suppression outdoors (~30 min)	18	No data	No data	Visual and auditory PASAT and CPT to measure information processing, working memory, and sustained attention	Impaired performance
Abrard et al. (2021)	Live-fire training exercise indoors (~30 min)	12	+400°C	37.3°C (Skin temperature)	Number subtraction task to measure attention and mental calculation	No effect
Canetti et al. (2022)	Live-fire training exercise indoors (15 min)	7	+400°C	38.9°C (Tympanic temperature)	Tasks to measure speed and accuracy, logical reasoning, and memory recall	No effect

## 2 Extreme heat improves cognition

Early research assessing the impact of heat on firefighter cognition found improvements following live-fire activities. In a study by Greenlee et al. (2014) firefighters completed a continuous performance test (CPT) to measure sustained attention before and after an 18-min indoor live-fire training scenario. Despite environmental temperatures reaching 82°C and core body temperature increasing from a mean of 37.1°C pre-training to 37.8°C post-training (reported in the earlier work of Horn et al., 2011), reaction times were faster post-training, demonstrating improved task performance after exposure to extreme temperatures.

The Maximal Adaptability Model suggests that stress initially enhances performance (arousal), but as stress levels increase and compensation is not possible, performance starts to decline. In the study of Greenlee and colleagues, it may be that heat stress was insufficient to compete for resources (i.e., due to temperatures not being extreme enough, core body temperature not exceeding critical levels identified by Schmit et al., 2017, and a short duration of heat exposure). However, Walker et al. (2015) also found improved performance following an indoor training exercise when firefighters were exposed to temperatures up to 115°C (core body temperatures reached 41°C) for 40-min. Participants completed search and rescue scenarios in the extreme temperatures and the researchers measured speed of processing, vigilance, and working memory before and after the activity. While they found no difference in performance pre- and post-activity for speed of processing and working memory, consistent with Greenlee et al. (2014) they found improved vigilance.

Given the temperatures used by Walker et al. (2015) it seems unlikely that improved performance can be attributed to an arousal effect. However, methodological drawbacks prevent any firm conclusion regarding this. When comparing performance on the same cognitive tests before and after heat exposure it is important to use a control condition/group as a comparison. Without this it is impossible to conclude that changes are not due to practice. The above findings do however seem in direct contrast to studies in the wider field of heat stress and cognition that have made use of a control condition (e.g., Liu et al., 2013; Qian et al., 2015), suggesting that firefighters are less impacted by heat stress than the general population. This could indicate some form of familiarization, or acclimatization, with firefighters better able to manage heat stress because they are more accustomed to it. In support of this, Radakovic et al. (2007) found that cognitive performance of soldiers was impaired after completing a heat stress test in 40°C compared to 20°C heat, unless they had been acclimatized to the heat for ten days.

## 3 Extreme heat impairs cognition

Evidence against firefighters being acclimatized to heat comes from studies showing heat negatively impacts cognition. Hemmatjo et al. (2017) tested cognition before and after an indoor firefighting scenario in low (29–31°C), moderate (32–34°C), and extreme heat (35–37°C) and measured information processing and working memory using a paced auditory serial addition task (PASAT). In this task participants hear numbers spoken one after the other and must add each number to the previous one. For example, hearing the numbers 8, 2, 5 participants should respond “10” after the second digit and “7” after the third digit. Hemmatjo et al. found decreased accuracy across all three conditions post-activity, although this was most pronounced in extreme heat.

In a later study, Hemmatjo et al. (2020) found similar effects after an outdoor training exercise. Firefighters passed through a fire in a large outdoor space, extinguished the fire using a water hose, and then turned off the hose. Before and after this they completed an auditory and visual PASAT, and an auditory and visual CPT. Although the researchers did not collect temperature measures, given the nature of the activity it would be assumed that participants were not exposed to the higher temperatures experienced in indoor training exercises (i.e., Walker et al., 2015), yet they found impaired performance across all tasks post-exercise.

This contrasts with the earlier work of Greenlee et al. (2014) who also used a visual CPT. However, the tasks used within each study were quite different. Greenlee et al. asked participants to monitor and respond to numbers, pressing Z for ‘frequent’ numbers (1–8, accounting for 80% of trials) and M for ‘rare’ numbers (0 and 9). Hemmatjo et al. presented participants with shapes and asked them to press a key when a star shape was shown (20% of trials) but make no response to other shapes. Since the task used by Hemmatjo and colleagues required participants to withhold a response on 80% of trials, it could be argued that it increased the chance of attention lapsing and is therefore a more suitable measure of sustained attention. Related to this, Walker et al. (2015) measured vigilance by asking participants to respond to the colour of playing cards, and measured working memory by asking whether each card matched the previous one. These tests seem more akin to simple perceptual judgment tasks than tasks such as the PASAT (Tombaugh, 2006), that measure information processing capacity. Greenlee et al. (2014) acknowledged the simplicity of their task and recommended investigating the effects of heat on more complex tasks, however an additional limitation that may be raised for all these studies is that there is no measurement of change in performance over time-on-task. This would be a better indicator of sustained attention for future studies, regardless of task complexity.

Zare et al. (2018) also used a PASAT before and after firefighters engaged in a live-fire training exercise outdoors. The exercise was the same as that used by Hemmatjo et al. (2020) and they compared this to two other scenarios; typical indoor training activities (carrying and pulling a hose, carrying and climbing a ladder, passing through unfamiliar narrow spaces, and passing through an escape tunnel) and rescue from height (a victim is suspended from the ceiling of a training room and a firefighter must use special ropes attached to the ceiling to lift themselves up to the victim and use a rescue belt to bring the victim down). Again, they found impairments following the live-fire training exercise. Interestingly, cognitive performance was worse post-exercise across all conditions and maximum core body temperature was also similar (38.07°C, 38.19°C, and 39°C for live-fire, typical training, and rescue from height respectively). The researchers found the greatest impairment in the rescue from height condition indicating that other sources of stress (e.g., physical fatigue, anxiety associated with rescuing a victim) will interact with heat stress to have a greater impact on cognition. These findings could be explained by the Global Workspace theory (Baars, 1997), which argues that stimuli compete for limited-capacity resources, and increased competition (i.e., from multiple sources of stress), puts more strain on cognitive resources, making it more likely that performance will be affected.

## 4 Extreme heat has no impact on cognition

The studies showing impairments to cognition are those that exposed firefighters to relatively moderate temperatures. It seems counterintuitive that more extreme temperatures would lead to no effect, or to improvements (as in Walker et al., 2015 and Greenlee et al., 2014), yet two studies that involved indoor live-fire training exercises with temperatures exceeding 400°C found no evidence that heat stress affects cognition. Abrard et al. (2021) measured cognition before and after firefighters experienced a live-fire exercise in a shipping container. Using a cognitive test from the Mini Mental State examination (Folstein et al., 1975) requiring participants to count backwards from 100 by 6, 7, or 8 they found no difference in performance pre- and post-exercise.

Canetti et al. (2022) also measured cognition before and after a live container fire exercise (temperatures exceeded 400°C) and found no differences. They assessed cognition with three tests; a digit cancelation task in which participants had 90-s to cross out targets on a sheet of paper, a logical reasoning task in which they had 30-s to answer true or false to statements about letter pairings (see Baddeley, 1968), and a recall task in which they were presented with items for 30-s and had to recall as many as possible.

These two studies had small sample sizes making it impossible to draw definitive conclusions, and while they report temperatures of over 400°C, environmental temperatures in a live-fire scenario can vary significantly. Abrard et al. report temperatures of 25°C-150°C at the back of the training structure, to over 450°C at helmet height, so perhaps participants were not exposed to such extreme temperatures. This would explain the relatively low maximum body temperatures recorded from participants (see Table 1) in comparison to the study completed by Walker et al. (2015). However, a key difference between these studies and those reporting negative effects of heat is the tasks used to assess cognition. The tasks used by Canetti et al. and Abrard et al. did not involve speeded responses, and crucially they did not measure processes that are most affected by heat stress such as sustained attention and working memory (Hancock and Vasmatzidis, 2003). Tasks that are easy will not draw heavily on limited cognitive resources, therefore any competition in the form of heat stress will have minimal impact on performance. This would explain why exposure to temperatures of 400°C+ did not impair cognition, but it raises questions over the effect of such temperatures on more complex cognitive functioning, and the suitability of tasks used to assess firefighter cognition.

## 5 Discussion

Evidence suggests that acute heat stress affects cognitive processing, with impairments found when environmental temperatures exceed 23°C (Ramsey et al., 1983) and core body temperatures exceed 39°C (Schmit et al., 2017). Past research also reveals that task complexity plays a significant role in the effects of heat, with complex functioning (e.g., Saini et al., 2017) impaired to a greater extent than simple functioning (e.g., Ashworth et al., 2021). Hancock and Vasmatzidis (2003) attribute this to competition for limited-capacity cognitive resources; heat stress puts a strain on resources, and while simple tasks are less resource-intensive and not impacted by this, competition in more complex situations will reduce available resources, impairing cognition.

Given the evidence from non-firefighter populations and the theoretical explanations for the effects of heat stress, it would be predicted that firefighters are at risk of cognitive impairments. Not only are they routinely exposed to working temperatures over 200°C (Willi et al., 2016), but search and rescue operations require complex cognitive functioning (Greenlee et al., 2014). Yet studies that measure the effects of heat on firefighter cognition show mixed findings. This review summarized seven research studies that measured cognitive functioning before and after firefighters completed a training exercise in high temperatures. Two studies showed improvements (Greenlee et al., 2014; Walker et al., 2015), three showed impairments (Hemmatjo et al., 2017; Zare et al., 2018; Hemmatjo et al., 2020), and two showed no effect of heat (Abrard et al., 2021; Canetti et al., 2022).

Across these studies there was no clear pattern in terms of the temperatures experienced (studies reporting the lowest temperatures found negative impacts), the duration of exposure (the longest exposure led to improved cognition), or the type of training exercise (varying effects were found in both indoor and outdoor activities). Added to this, the varying cognitive tasks used makes comparison across the different studies difficult. However, in accordance with research from non-firefighter populations, the one consistent feature was that acute heat stress had a negative effect on more complex functioning. Using the classification of Taylor et al. (2016), the work showing no effect of heat, or improvements, arguably tested “simple” functions (choice reaction, memory recall, and simple arithmetic) and those showing negative effects of heat tested “complex” functions (vigilance, sustained attention, and working memory). This aligns with research by Gaoua et al. (2018) showing heat stress is a source of cognitive load that affects activity in the frontal cortex, and while this does not affect completion of simple tasks, competition for resources means that performance suffers in complex tasks. This is supported by the model of Hermans et al. (2014) that stressors cause reduced activity in the prefrontal cortex to allow increased activity in other areas of the brain.

While it is argued that the varied findings presented here can be attributed to the different effects of heat stress on different cognitive functions, there are other factors that may play a role. For instance, hydration status, extent of physical activity, and the requirement to wear personal protective equipment have all been shown to affect cognition (e.g., Tomporowski, 2003; Masento et al., 2014; Park, 2019) and may therefore moderate the direct impact of heat stress. Some studies provide clear details about equipment worn during activity, physical exertion rates, hydration status, etc., but many do not. Without this it is difficult to identify the exact effects of heat on cognition in a fire and rescue scenario, and future work should endeavor to collect and report such information.

Differences between studies measuring the effects of heat stress in the general population and studies measuring the effects of heat stress in firefighters also make it difficult to fully assess the impact of heat on firefighter cognition. Firefighters are exposed to much higher temperatures therefore research in the general population does not reflect the working conditions of firefighters. Also, most studies conducted with the general population make effective use of control groups, and they test cognition concurrent to heat exposure, rather than after heat exposure. Despite these differences, this short review suggests that the effects of acute heat stress on firefighters are like those found in the general population; specifically acute heat stress impairs complex cognition but not simple cognition.

This conclusion is concerning because search and rescue operations require complex functioning and by measuring simplistic processing, some existing research does not show the true extent of heat stress on firefighters. Future work should therefore make use of tasks that better reflect the cognitive demands of firefighting. In addition, the limited research using firefighters provides minimal information about factors that moderate the effects of heat stress, such as exertion levels, hydration, expertise, and acclimatization (Hancock and Vasmatzidis, 2003). Therefore, while this research has the potential to inform operational guidelines and working practices in relation to how long firefighters can work in extreme temperatures and how long they should spend cooling, more work is needed to gain a full understanding of the risks of acute heat stress on firefighter cognition. Future work using the recommendations outlined here would benefit firefighters but would also apply to other occupational groups for which heat exposure may be a hazard, such as military personnel and construction workers.

## Author contributions

CT: Conceptualization, Funding acquisition, Writing – original draft, Writing – review & editing. LF: Writing – original draft. SP: Funding acquisition, Writing – review & editing. BH: Writing – review & editing. MM: Funding acquisition, Writing – review & editing.
